# Factors associated with junior doctors’ decisions to apply for general practice training programmes in the UK: secondary analysis of data from the UKMED project

**DOI:** 10.1186/s12916-017-0982-6

**Published:** 2017-12-21

**Authors:** Thomas C. E. Gale, Paul J. Lambe, Martin J. Roberts

**Affiliations:** 1 Collaboration for the Advancement of Medical Education Research and Assessment (CAMERA), Plymouth University Peninsula Schools of Medicine and Dentistry, John Bull Building, Plymouth Science Park, Plymouth, PL6 8BU UK; 2 Plymouth Hospitals NHS Trust, Derriford Rd, Plymouth, PL6 8DH UK

**Keywords:** Medical education, General practice, Primary care, Recruitment, Workforce planning

## Abstract

**Background:**

The UK, like many high-income countries, is experiencing a worsening shortfall of general practitioners (GPs) alongside an increasing demand for their services. At the same time, factors influencing junior doctors’ decisions to apply for GP training are only partially understood and research in this area has been hampered by the difficulties in connecting the datasets that map the journey from student to qualified GP. The UK Medical Education Database (UKMED) has been established to ameliorate this problem by linking institutional data across the spectrum of medical education from school to specialty training. Our study aimed to use UKMED to investigate which demographic and educational factors are associated with junior doctors’ decisions to apply for GP training.

**Methods:**

Study data, provided by the UKMED Development Group and accessed remotely, contained longitudinal educational and sociodemographic information on all doctors who entered UK medical schools in the 2007/2008 academic year and who made first-time specialty training applications in 2015. We used multivariable logistic regression models to investigate two binary outcomes, namely (1) application to GP training, possibly alongside applications to other specialties, and (2) application solely to GP training.

**Results:**

Of 7634 doctors in the sample, 43% applied to GP training possibly alongside other specialities and 26% applied solely to GP training. The odds of applying to GP training were associated with particular demographic factors (being female, non-white or secondary educated in the UK increased the odds of application) and educational factors (non-graduate entry, intercalation and above-median academic performance during medical school all decreased the odds of application). After adjusting for these factors, both the medical school and the foundation school attended were independently associated with the odds of applying to GP training.

**Conclusions:**

Our findings suggest that the supply and demand imbalance in UK primary care might be improved by (1) efforts to attract greater numbers of female, non-white and UK secondary-educated students into medical schools, and (2) targeting resources at medical and foundation schools that deliver doctors likely to fill significant gaps in the workforce. Further research is required to better understand inter-school differences and to develop strategies to improve recruitment of GP trainees.

**Electronic supplementary material:**

The online version of this article (doi:10.1186/s12916-017-0982-6) contains supplementary material, which is available to authorized users.

## Background

There are significant concerns worldwide regarding the number of general practitioners (GPs) available to meet the demands of aging and increasingly complex healthcare populations, with trends over the last 20 years in many high-income countries, including the UK, signifying lower percentages of GPs in the total medical workforce [1, 2]. Workforce planning reports in the US and UK project worsening shortfalls of primary care providers towards 2030, with a major demand–supply imbalance [3, 4]. In response, the UK Department of Health issued a mandate to Health Education England to recruit 50% of foundation doctors to general practice (GP) training programmes by 2016 [5, 6]. However, despite recruitment initiatives [7], only 33.8% of the pool of successful applicants to specialty training were appointed to GP in 2016 compared to 36.1% in 2012 [8]. Further, long-term trends consistently show that only approximately 36% of each UK cohort enter the GP register [9].

Research into the factors associated with medical students’ specialty choice has indicated that decisions to apply for postgraduate training programmes are shaped by a multiplicity of factors and that medical career decision-making is a dynamic and complex process that is not yet fully understood [9, 10]. Curricular and institutional cultural biases against primary care may exist in medical schools and postgraduate training placements, which could influence subsequent career choice [11], but variation in entry to GP training across UK medical schools remains relatively unexplored [12]. Moreover, early clinical placements, longitudinal integrated clerkships and positive role models in primary care are thought to impact positively on intentions to pursue a career in GP [13]. Survey-based research in a number of countries has focused on identifying both the individual characteristics (sociodemographic, academic and attitudinal) and institutional factors that are associated with self-reported (intended) career preferences of both medical students and early-career doctors [14–20]. However, intentions to follow a particular career pathway are subject to change and may not materialise as actual applications to particular specialty training programmes [15, 21]. Nevertheless, a recent evaluation of GP selection in the UK showed intentions to be predictive of doctors ending up on the GP register, with the strength of association increasing with the recency of the stated intention [9]. Importantly, most extant research has only examined the association between career choice and one or a few variables [10]. Further work is needed to evaluate the extent to which independent factors are associated with actual applications to GP training, to differentiate their relative strengths, and to detect interrelationships between hypothesized predictors.

The UK Medical Education Database (UKMED) has recently been established to provide a secure repository of longitudinal data related to the performance of UK medical students and trainee doctors [22]. The dataset contains information on sociodemographic factors and measures of academic attainment from secondary school through to postgraduate training, as well as choices made by individuals with respect to undergraduate training, foundation doctor placements and applications to specialty training. This database provides a unique and valuable opportunity to add to the literature on career choice by permitting analysis, for a large cohort of UK doctors, of some of the important factors that are associated with the decision to apply for a place on the nationally recruited GP specialty training programme. The primary aim of this study was to identify significant independent factors associated with the decision to apply for GP training and to delineate typologies of applicants likely to apply. This information is of value to policymakers and educationalists involved with attempts to increase the proportion of GPs in the medical workforce.

## Methods

### Data, study population and variables

Anonymised data for this study was provided by the UKMED Development Group and accessed remotely by the authors via the Health Informatics Centre Safe Haven at Dundee University (https://www.dundee.ac.uk/hic/hicsafehaven/). The UKMED Data Dictionary describes the available data that provided longitudinal educational and sociodemographic information on all students entering UK medical schools in the 2007/2008 academic year [22]. The study population comprised all doctors who applied for specialty training in 2015 and for whom there was no prior application record in the UKMED data, thereby focussing on ‘first time’ applications to specialty training. Application data for earlier and later years was not, at the time of the study, available in UKMED.

We used multivariable logistic regression models to investigate two binary outcomes, namely (1) whether or not the doctor applied to GP specialty training, possibly alongside applications to other specialties, and (2) whether or not the doctor applied solely to GP specialty training.

Independent variables included a range of background factors such as personal, family, academic, medical school, and foundation school (see Additional file 1: Table S1 for distributions and missing values and the UKMED Data Dictionary at http://www.ukmed.ac.uk/documents/UKMED_data_dictionary.pdf for a full list of data types, descriptions and sources).

### Statistical analysis

We conducted univariate analyses to identify missing, unexpected and outlying values, and to assess the data for normality of distribution. For each outcome, we used bivariate tests of association with each independent variable (Fisher’s Exact Test, Pearson’s χ^2^ test and univariate logistic regression, as appropriate) to inform the construction of multivariable logistic regression models. List-wise deletion excluded cases in which there were missing values for any of the variables in the regression model.

We categorised doctors’ medical school Entry Status as either ‘Graduate entrant to Standard Programme’ , ‘Non-graduate entrant to Standard Programme’ or ‘Entrant to Graduate Programme’. Because data are not widely available for graduate entrants to medical school on the total Higher Education Statistics Agency (HESA) tariff (a score for qualifications held on application to medical school) and total UK Clinical Aptitude Test (UKCAT) score, we conducted analyses of the two outcomes on all doctors (Sample A; n = 7634) and doctors who had been non-graduate entrants to their medical degree programmes (Sample B; n = 5540).

In respect of Sample A and outcomes 1 and 2, preliminary multivariable models revealed the Index of Multiple Deprivation (IMD, a neighbourhood-based measure of social deprivation), the participation of local areas (POLAR2) classification (a neighbourhood-based measure of participation in Higher Education) and type of secondary school attended, to be non-significant. Along with total HESA tariff and total UKCAT score, these three variables were eliminated from the two final models.

In respect of Sample B and outcome 1, a preliminary multivariable model revealed type of secondary school attended, POLAR2, National Statistics Socio-economic Classification (NS-SEC), medical school entry status and total HESA tariff to be non-significant and they were eliminated from this model.

In respect of Sample B and outcome 2, a preliminary multivariable model revealed IMD, POLAR2, NS-SEC, entry status, UK secondary educated, total HESA Tariff, educational performance measure (EPM, a rank-based indicator of attainment during medical school) and the most recent Annual Review of Clinical Progression (ARCP, an indicator of successful progress through postgraduate foundation training) to be non-significant and they were eliminated from this model.

The variables age at entry to medical school, parent degree (whether either parent was a university graduate), income support, and free-school meals (both indicators of low socioeconomic status during schooling) failed to reach significance in any of the preliminary multivariable models and were excluded from all four final models.

We assessed model goodness-of-fit using the Hosmer–Lemeshow test with a *P* value greater than 0.05 taken to indicate acceptable fit [23]. The significance of the effect of individual predictor variables was assessed using a z-test (Wald Test computed as a χ^2^ test) with a *P* value less than 0.05 taken to indicate statistical significance [24]. The quality of model classification (sensitivity and specificity of predicted outcomes) was assessed using receiver operating characteristic diagnostics [25]. The adequacy of model sample size was assessed using the formula N = 10 x k/p, where p is the proportion of negative or positive cases (whichever smallest) in the population and k is the number of independent variables, to indicate the minimum number of cases required [26].

We examined interaction effects and finally we interpreted the modelling results in relation to the aims of the study, basing our interpretations on predicted probabilities. Typologies, based on profiles of values for the independent variables in a model, enabled insight into which configuration of variables were substantively important in influencing the outcome [24]. We used Stata 14 for all analyses.

## Results

### Descriptive statistics

The majority of doctors in the sample (70%; 5390/7634) applied to a single specialty, while 99% (7551/7634) applied to three or less specialties (Table 1). Overall, 43% (3307/7634) applied to GP specialty training and 26% (1954/7634) applied solely to GP specialty training. Among those applying to multiple specialties, the majority (60%; 1353/2244) applied to GP training. Rates of application to GP specialty training varied by the medical school (25–60%) and by the foundation school (32–56%) that doctors had attended (Additional file 2: Table S2).

**Table 1:** Frequency and patterns of specialty training applications (n = 7634)

Number of specialty applications	Number of doctors	Percentage of sample	Applied to GP specialty training	
			n	Percentage within number of applications
1	5390	70.61	1954	36.25
2	1812	23.74	1022	56.40
3	349	4.57	260	74.50
4–9	83	1.09	71	85.54
All	7634	100	3307	43.32
Percentage of whole sample that applied to GP specialty training			43.32% (3307/7634)	
Percentage of whole sample that applied solely to GP training			25.60% (1954/7634)	

### Bivariate analyses

Bivariate tests of association revealed statistically significant associations between the outcome (having applied to GP specialty training) and many of the independent variables (Additional file 1: Table S1, final column). Significant variables were included in exploratory multivariable logistic regression models.

### Model 1: Applied to GP specialty training (all entry programmes)

The analytic sample (n = 6177) comprised doctors who had graduated from all types of medical school programmes, classified as non-graduate entrants to standard entry programmes (SE, n = 5008), graduate entrants to standard entry programmes (GSE, n = 668) and entrants to graduate entry programmes (GE, n = 501).

A Hosmer–Lemeshow test confirmed adequate model fit and Wald tests confirmed that sex, Black and minority ethnic group (BME), UK secondary education, entry status, intercalation, medical school attended, EPM and ARCP were significantly associated with having applied to GP specialty training, whilst NS-SEC and foundation school were non-significant (Table 2). The interaction of BME and intercalation was significant (χ^2^ = 7.01, df = 1, *P* < 0.01) as was the interaction of BME and medical school entry status (χ^2^ = 7.25, df = 2, *P* < 0.05).

**Table 2:** Results of logistic regression modelling

		Outcome 1				Outcome 2			
		Applied to GP training				Applied solely to GP training			
		Model 1 Sample A All medical school entrants		Model 2 Sample B Non-graduate entrants only		Model 3 Sample A All medical school entrants		Model 4 Sample B Non-graduate entrants only	
Predictor	df	χ^2^	*P* value	χ^2^	*P* value	χ^2^	*P* value	χ^2^	*P* value
Sex	1	76.37	0.0000	66.71	0.0000	43.87	0.0000	37.04	0.0000
BME	1	31.81	0.0000	22.92	0.0000	n/s	n/s	9.05	0.0026
NS-SEC	4	n/s	n/s			13.27	0.0100		
IMD	4			10.95	0.0272				
POLAR2	2								
Secondary school type	1							3.66	0.0558
UK secondary educated	1	16.43	0.0000	n/s	n/s	8.10	0.0044		
Total UKCAT z-score	1			16.87	0.0000			5.44	0.0197
Total HESA tariff z-score	1								
Medical school entry status	2	9.73	0.0077			6.25	0.0440		
Medical school	32	91.56	0.0000	83.18	0.0000	79.78	0.0000	76.17	0.0000
Intercalated	1	36.03	0.0000	40.89	0.0000	17.63	0.0000	22.61	0.0000
EPM top two quartiles	1	6.85	0.0088	4.50	0.0339	n/s	n/s		
Foundation school	26	n/s	n/s	44.43	0.0136	47.88	0.0056	45.07	0.0116
ARCP satisfactory progression	1	5.07	0.0243	n/s	n/s	n/s	n/s		
Model statistics									
Minimum required sample size		234		232		392		303	
Actual sample size		6177		4327		6177		4375	
Mean probability (95% CI)		0.427 (0.416 to 0.440)		0.431 (0.417 to 0.445)		0.255 (0.245 to 0.266)		0.264 (0.256 to 0.281)	
Hosmer–Lemeshow test		χ^2^(8) = 10.03 *P* = 0.263		χ^2^(8) = 7.43 *P* = 0.491		χ^2^(8) = 6.09 *P* = 0.637		χ^2^(8) = 10.45 *P* = 0.235	
Area under ROC curve		0.6421		0.6698		0.6394		0.6603	

Blank cells indicate non-significant predictor in preliminary model and excluded from final model

*ARCP* Annual Review of Clinical Progression, *BME* Black and minority ethnic, *CI* confidence interval, *EPM* Educational Performance Measure, *HESA* Higher Education Statistics Agency, *IMD* Index of Multiple Deprivation, n/s non-significant predictor in reported final model, *NS*-*SEC* National Statistics Socio-economic Classification, *POLAR2* Participation Of Local Areas classification, *ROC* receiver operating characteristic, *UKCAT* UK Clinical Aptitude Test

Being from a BME background, UK secondary educated or progressing satisfactorily at ARCP were associated with higher odds of having applied to GP training, while being male, intercalating during medical school or being placed in the top two EPM quartiles were associated with lower odds. Although the effect of entry status on the odds of application was significant, the only significant difference was between GSE (reference category) and SE (Table 3).

**Table 3:** Odds ratios (OR) and associated 95% confidence intervals for independent predictors of the outcomes in logistic regression models 1 to 4

Factor	Model 1	Model 2	Model 3	Model 4
Male	0.62 (0.55 to 0.69)	0.58 (0.51 to 0.66)	0.66 (0.58 to 0.74)	0.63 (0.55 to 0.73)
BME	1.43 (1.27 to 1.63)	1.48 (1.26 to 1.74)		1.31 (1.1 to 1.57)
Independent/Private school				0.85 (0.72 to 1.00)
UK secondary educated	1.92 (1.40 to 2.63)		1.73 (1.19 to 2.53)	
IMD Quintile 1		0.66 (0.50 to 0.86)		
IMD Quintile 2		0.77 (0.58 to 1.02)		
IMD Quintile 3		0.74 (0.55 to 0.98)		
IMD Quintile 4		0.73 (0.54 to 0.99)		
IMD Quintile 5		1 (Reference)		
UKCAT z-score		0.86 (0.80 to 0.92)		0.91 (0.84 to 0.98)
NS-SEC1 Routine/semi-routine			1 (Reference)	
NS-SEC2 Lower supervisory/technical			0.70 (0.44 to 1.12)	
NS-SEC3 Small employer/own account			0.91 (0.67 to 1.23)	
NS-SEC4 Intermediate			0.74 (0.57 to 0.96)	
NS-SEC5 Managerial/Professional			0.72 (0.60 to 0.88)	
Entry Status: Graduate entrant to standard programme	1 (Reference)		1 (Reference)	
Entry Status: Non-graduate entrant to standard programme	0.76 (0.63 to 0.91)		0.80 (0.65 to 0.97)	
Entry Status: Graduate programme	0.87 (0.65 to 1.17)		0.70 (0.51 to 0.98)	
MS: Aberdeen	3.35 (1.93 to 5.82)	3.25 (1.59 to 6.66)	7.45 (2.76 to 20.06)	4.83 (1.67 to 13.95)
MS: Barts	3.44 (2.16 to 5.48)	6.09 (3.31 to 11.21)	8.05 (3.11 to 20.83)	7.60 (2.83 to 20.40)
MS: Birmingham	2.28 (1.43 to 3.64)	2.42 (1.35 to 4.35)	5.07 (1.96 to 13.16)	3.52 (1.32 to 9.38)
MS: Bradford^a^	7.11 (2.74 to 18.47)	6.24 (1.93 to 20.14)	22.08 (6.39 to 76.26)	13.24 (3.33 to 52.6)
MS: Brighton Sussex	2.83 (1.64 to 4.88)	3.87 (1.97 to 7.6)	5.59 (2.05 to 15.25)	4.62 (1.62 to 13.15)
MS: Bristol	2.07 (1.25 to 3.43)	2.41 (1.26 to 4.6)	5.52 (2.09 to 14.58)	3.62 (1.30 to 10.04)
MS: Cambridge	1 (Reference)	1 (Reference)	2.32 (0.83 to 6.53)	1.41 (0.46 to 4.28)
MS: Cardiff	2.60 (1.57 to 4.32)	3.60 (1.93 to 6.71)	5.73 (2.17 to 15.16)	5.18 (1.91 to 14.07)
MS: Dundee	2.49 (1.39 to 4.45)	2.59 (1.20 to 5.60)	4.29 (1.53 to 12.04)	3.73 (1.22 to 11.39)
MS: Edinburgh	1.70 (1.00 to 2.90)	2.15 (1.10 to 4.20)	3.82 (1.41 to 10.33)	3.72 (1.33 to 10.38)
MS: Glasgow	2.78 (1.62 to 4.76)	2.88 (1.42 to 5.86)	5.66 (2.10 to 15.27)	4.16 (1.44 to 12.01)
MS: Hull York	4.74 (2.78 to 8.08)	7.36 (3.70 to 14.62)	8.67 (3.24 to 23.17)	8.21 (2.92 to 23.04)
MS: Imperial	1.70 (1.04 to 2.78)	2.08 (1.13 to 3.83)	3.61 (1.34 to 9.71)	2.37 (0.85 to 6.60)
MS: Keele	2.35 (1.38 to 3.99)	2.02 (1.04 to 3.91)	4.59 (1.69 to 12.43)	2.96 (1.04 to 8.39)
MS: King’s	2.03 (1.24 to 3.34)	3.09 (1.58 to 6.04)	5.38 (2.03 to 14.24)	3.76 (1.30 to 10.90)
MS: Lancaster	2.58 (1.28 to 5.22)	2.00 (0.81 to 4.97)	7.05 (2.35 to 21.16)	2.67 (0.75 to 9.50)
MS: Leeds	2.90 (1.67 to 5.04)	3.62 (1.81 to 7.23)	5.89 (2.15 to 16.15)	4.41 (1.53 to 12.74)
MS: Leicester	3.14 (1.92 to 5.14)	2.98 (1.60 to 5.55)	6.69 (2.55 to 17.51)	3.63 (1.33 to 9.90)
MS: Liverpool	3.51 (2.14 to 5.77)	3.45 (1.85 to 6.41)	6.43 (2.44 to 16.95)	4.04 (1.49 to 10.96)
MS: Manchester	3.04 (1.88 to 4.92)	3.46 (1.90 to 6.32)	6.12 (2.34 to 15.97)	4.47 (1.67 to 12.00)
MS: Newcastle	3.67 (2.21 to 6.10)	4.82 (2.54 to 9.13)	6.50 (2.44 to 17.31)	4.91 (1.77 to 13.61)
MS: Norwich	2.20 (1.35 to 3.61)	2.61 (1.38 to 4.93)	5.07 (1.91 to 13.44)	3.24 (1.16 to 9.03)
MS: Nottingham	2.14 (1.34 to 3.41)	2.64 (1.46 to 4.77)	5.83 (2.25 to 15.15)	4.48 (1.68 to 11.95)
MS: Oxford	1.11 (0.59 to 2.07)	1.48 (0.71 to 3.12)	1 (Reference)	1 (Reference)
MS: Peninsula	3.37 (2.01 to 5.63)	3.64 (1.92 to 6.91)	8.82 (3.34 to 23.31)	6.27 (2.30 to 17.14)
MS: Queen’s Belfast	3.38 (1.75 to 6.52)	3.34 (1.51 to 7.37)	4.71 (1.57 to 14.18)	3.57 (1.12 to 11.38)
MS: Sheffield	3.59 (2.07 to 6.23)	4.41 (2.26 to 8.59)	8.74 (3.22 to 23.71)	6.82 (2.44 to 19.03)
MS: Southampton	2.50 (1.52 to 4.09)	2.6 (1.39 to 4.86)	4.75 (1.80 to 12.57)	3.85 (1.41 to 10.53)
MS: St Andrews	2.55 (1.32 to 4.92)	2.13 (0.93 to 4.86)	4.44 (1.48 to 13.30)	1.53 (0.42 to 5.52)
MS: St George’s	3.11 (1.91 to 5.06)	4.03 (2.12 to 7.67)	7.46 (2.84 to 19.62)	7.04 (2.55 to 19.42)
MS: Swansea	3.34 (1.58 to 7.04)		11.27 (3.64 to 34.86)	
MS: University College London	2.06 (1.19 to 3.55)	3.39 (1.70 to 6.77)	4.85 (1.75 to 13.43)	4.97 (1.70 to 14.52)
MS: Warwick	2.24 (1.22 to 4.11)		6.71 (2.34 to 19.29)	
Intercalated	0.56 (0.46 to 0.68)	0.49 (0.39 to 0.61)	0.62 (0.50 to 0.78)	0.55 (0.43 to 0.70)
EPM top two quartiles	0.86 (0.77 to 0.96)	0.86 (0.75 to 0.99)		
FS: Birmingham		2.22 (1.26 to 3.92)	1.65 (0.95 to 2.87)	3.49 (1.14 to 10.68)
FS: Black Country/Shropshire		1.91 (1.06 to 3.45)	1.72 (0.97 to 3.03)	3.37 (1.09 to 10.47)
FS: Coventry/Warwickshire		2.00 (0.89 to 4.49)	1.35 (0.68 to 2.68)	1 (Reference)
FS: East Anglian		1.16 (0.67 to 2.01)	1.82 (1.09 to 3.04)	2.87 (0.94 to 8.78)
FS: Hereford/Worcestershire		1.19 (0.57 to 2.50)	1.40 (0.69 to 2.84)	2.55 (0.74 to 8.84)
FS: Leicester/Northamptonshire		2.15 (1.19 to 3.89)	2.46 (1.41 to 4.28)	4.47 (1.45 to 13.84)
FS: Mersey		1.10 (0.62 to 1.93)	1.67 (0.97 to 2.87)	3.18 (1.03 to 9.84)
FS: North Central Thames		1 (Reference)	1.06 (0.60 to 1.86)	1.48 (0.44 to 4.96)
FS: North East Thames		1.29 (0.72 to 2.30)	1.24 (0.73 to 2.12)	2.34 (0.74 to 7.35)
FS: North West Thames	1 (Reference)	1.02 (0.57 to 1.84)	1 (Reference)	2.41 (0.75 to 7.75)
FS: North Western		1.21 (0.72 to 2.03)	1.17 (0.71 to 1.93)	2.31 (0.77 to 6.95)
FS: North Yorkshire/E. Coast		0.87 (0.46 to 1.64)	1.35 (0.75 to 2.42)	3.01 (0.95 to 9.61)
FS: Northern		0.89 (0.51 to 1.55)	1.03 (0.60 to 1.79)	1.88 (0.61 to 5.83)
FS: Northern Ireland		1.67 (0.80 to 3.50)	1.07 (0.50 to 2.26)	1.94 (0.54 to 6.94)
FS: Oxford		0.98 (0.54 to 1.77)	1.21 (0.68 to 2.15)	2.30 (0.72 to 7.37)
FS: Peninsula		1.07 (0.57 to 2.01)	1.21 (0.67 to 2.19)	2.28 (0.71 to 7.33)
FS: Scotland E. Region		0.79 (0.34 to 1.81)	1.03 (0.47 to 2.25)	1.61 (0.42 to 6.17)
FS: Scotland N. Region		2.51 (1.25 to 5.05)	2.69 (1.45 to 5.00)	5.69 (1.72 to 18.79)
FS: Scotland W. Region		1.34 (0.74 to 2.45)	1.95 (1.12 to 3.38)	3.62 (1.15 to 11.4)
FS: Severn		1.23 (0.72 to 2.11)	1.72 (1.04 to 2.83)	3.46 (1.13 to 10.59)
FS: South Thames		1.26 (0.78 to 2.05)	1.24 (0.78 to 1.97)	2.30 (0.77 to 6.84)
FS: South Yorkshire		1.09 (0.57 to 2.07)	1.02 (0.54 to 1.92)	1.90 (0.58 to 6.21)
FS: Staffordshire		1.94 (1.02 to 3.68)	1.90 (1.02 to 3.54)	3.77 (1.19 to 12.00)
FS: Trent		1.43 (0.84 to 2.46)	1.70 (1.02 to 2.84)	3.73 (1.23 to 11.26)
FS: Wales		1.28 (0.73 to 2.24)	1.62 (0.95 to 2.76)	2.58 (0.84 to 7.95)
FS: Wessex		1.25 (0.72 to 2.16)	1.55 (0.92 to 2.63)	2.96 (0.97 to 9.06)
FS: West Yorkshire		1.05 (0.60 to 1.85)	1.50 (0.88 to 2.58)	2.94 (0.95 to 9.07)
ARCP Satisfactory progression	1.21 (1.03 to 1.44)			

^a^University of Bradford Foundation Course was classified by HESA as ‘medical school first attended’ by doctors in UKMED database

*ARCP* Annual Review of Clinical Progression, *BME* Black and minority ethnic, *EPM* Educational Performance Measure, *FS* foundation school, *IMD* Index of Multiple Deprivation, *MS* medical school, *NS*-*SEC* National Statistics Socio-economic Classification, *UKCAT* UK Clinical Aptitude Test

**Fig. 1:**
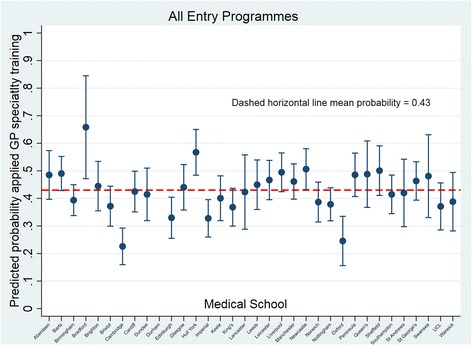
Predicted probability of having applied to GP specialty training contrasted by medical school attended (Model 1: sample = all entry programmes)

Variance among medical schools is clearly illustrated in Fig. 1. Compared to Cambridge (reference category), subjects who had studied at all other UK medical schools, Oxford and Edinburgh apart, were significantly more likely to have applied to GP specialty training. (Table 3).

#### Typologies

The mean predicted probability of having applied to GP specialty training for subjects on all entry programmes was 0.43 (Table 2). Irrespective of medical school entry status, BME females who had been UK secondary school educated and had not intercalated at medical school had the highest predicted probability of having applied to GP specialty training (SE = 0.55, GSE = 0.61, GE = 0.58) (Additional file 3: Table S3). In contrast, white male intercalaters whose secondary education was outside of the UK were the least likely (SE = 0.13, GSE = 0.17, GE = 0.15).

**Fig. 2:**
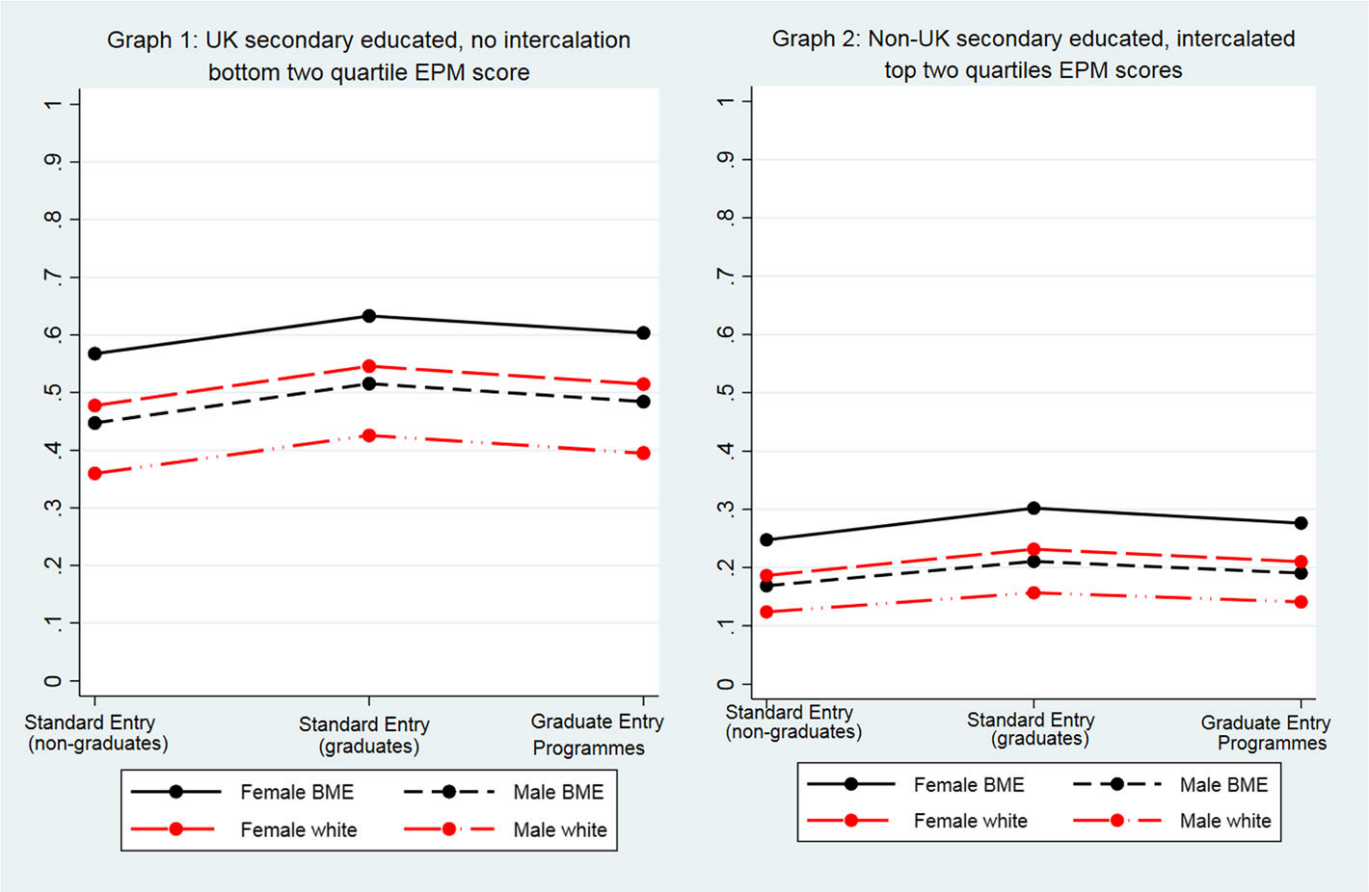
Predicted probability of having applied to GP specialty training adjusted by entry status, sex, BME, intercalation, UK secondary education and EPM (Model 1: sample = all entry programmes)

For any given typology, graduate entrants to standard entry programmes had a higher probability of having applied to GP specialty training than non-graduate entrants to standard entry programmes and those on graduate entry programmes (Additional file 3: Table S3) (Fig. 2).

### Model 2: Applied to GP specialty training (non-graduate entrants to medical school)

The analytic sample comprised subjects on standard entry programmes (n = 4327) excluding graduate entrants. Sex, BME, IMD, total UKCAT score, intercalation, medical school, foundation school and EPM were significantly associated with having applied to GP specialty training, whilst ARCP and UK secondary educated were non-significant (Table 2).

**Fig. 3:**
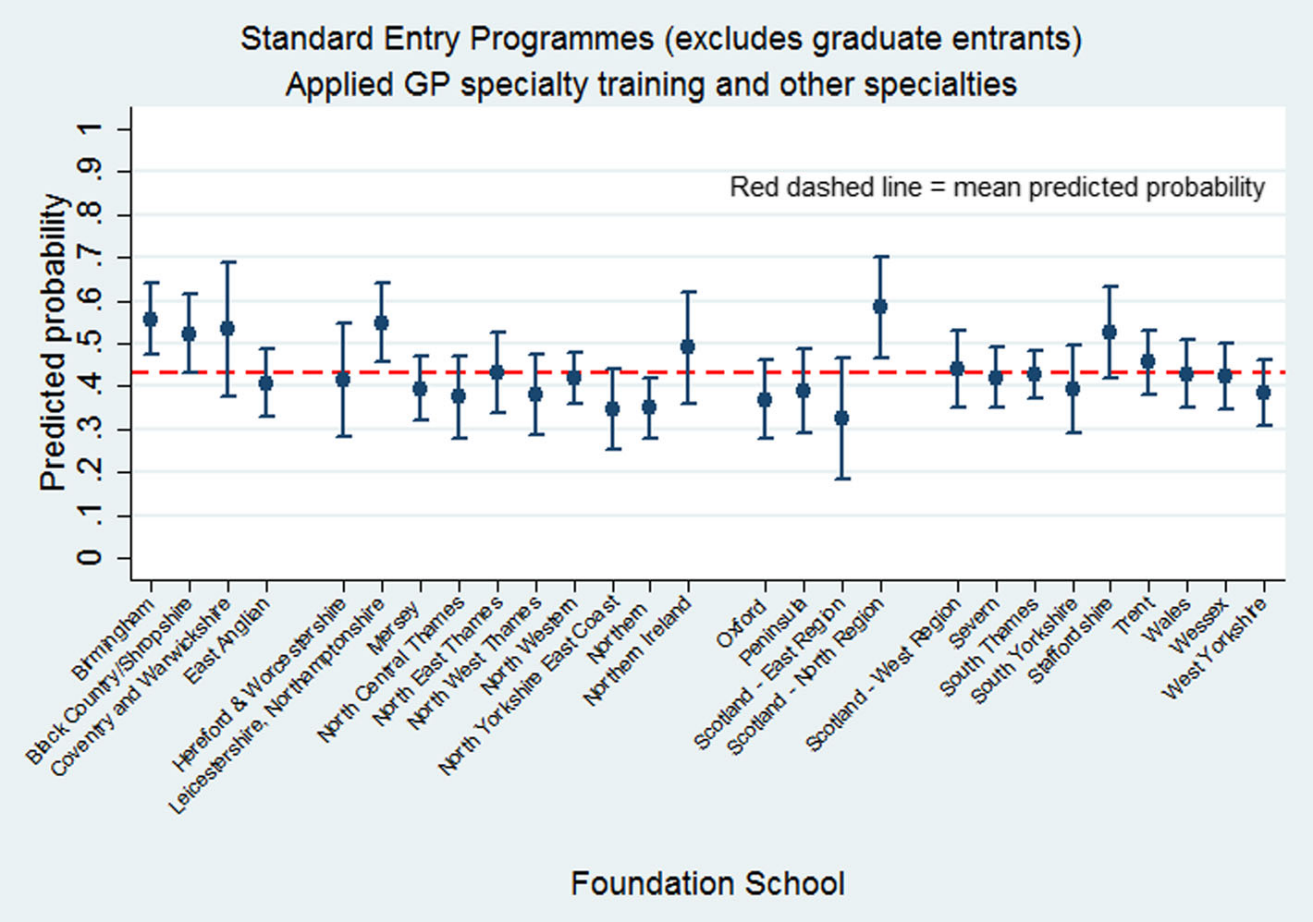
Predicted probability of having applied to GP specialty training contrasted by foundation school attended (Model 2; sample = standard entry programmes excluding graduate entrants)

Being from a BME background was associated with higher odds of having applied to GP training, while being male, coming from an area of low deprivation, having a high UKCAT score, intercalating during medical school or being placed in the top two EPM quartiles were associated with lower odds. The odds varied significantly among both medical schools and foundation schools (Table 3). Variation among foundation schools is shown in Fig. 3.

#### Typologies

**Fig. 4:**
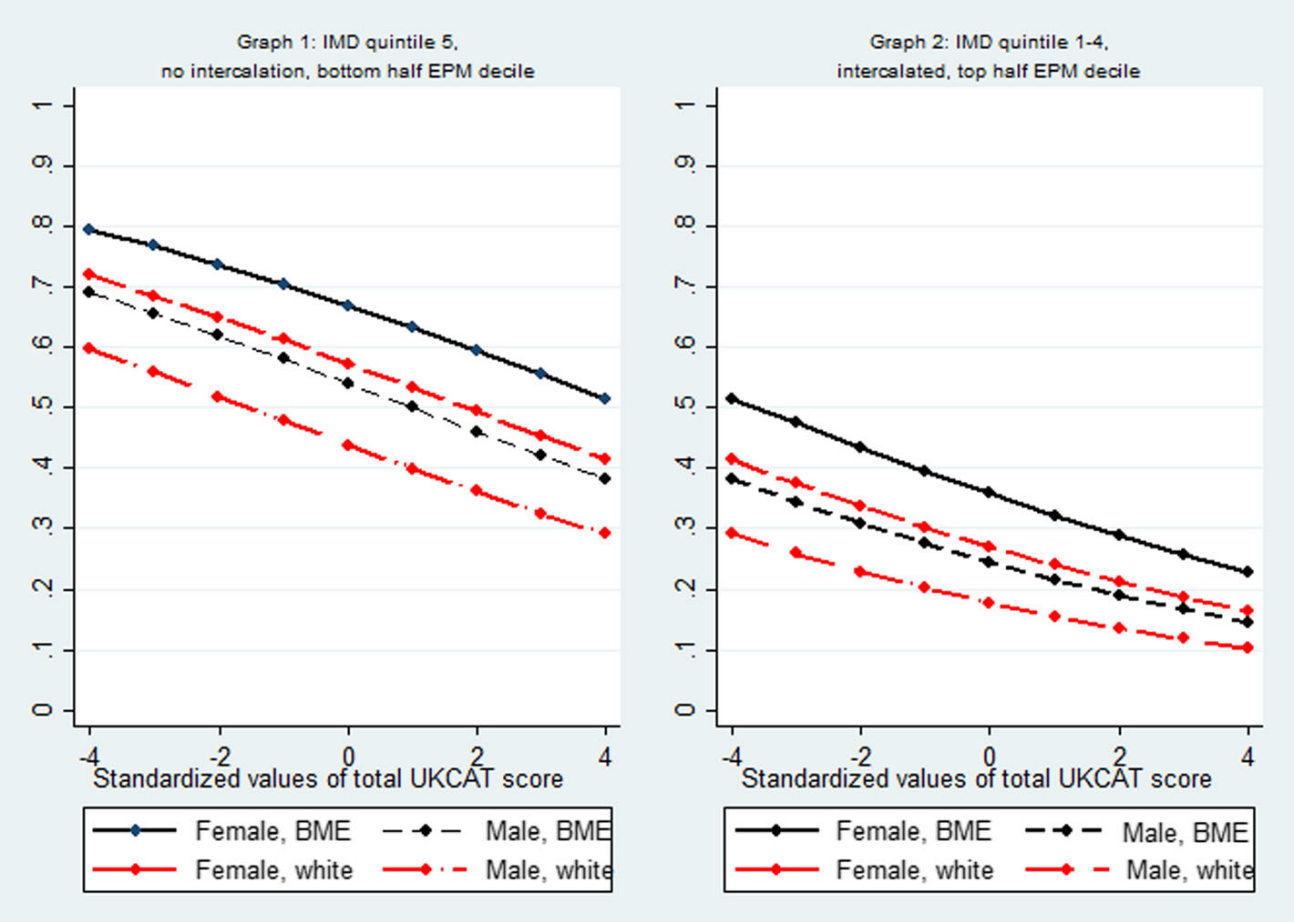
Predicted probability of having applied to GP specialty training adjusted by sex, BME, IMD, EPM and performance in the UK Clinical Aptitude Test (Model 2; sample = standard entry programmes, excluding graduate entrants)

The mean predicted probability of having applied to GP specialty training for subjects who had studied medicine as non-graduate entrants was 0.43 (Table 2). Female, BME subjects in IMD quintile 5 who had not intercalated and were not in the top two EPM quartiles had the highest probability of having applied to GP specialty training (0.67), while male, white doctors in IMD quintile 1 who had intercalated and scored in the top half of EPM deciles had the lowest probability (0.18) (Additional file 4: Table S4, Fig. 4). Moreover, as performance in the UKCAT increased, the probability of having applied to GP specialty training and other specialties decreased (Fig. 4).

### Model 3: Applied solely to GP specialty training (all entry programmes)

Overall, 25.6% of doctors applied solely to GP specialty training. Contrasted by medical school attended, this percentage ranged from 7% to 52% and by foundation school attended from 16% to 40% (Additional file 2: Table S2).

Sex, NS-SEC, UK secondary educated, entry status, intercalation, medical school and foundation school were significantly associated with the likelihood of having applied solely to GP specialty training, whilst BME, EPM and ARCP were non-significant (Table 2). The interaction of sex and NS-SEC was also significant (χ^2^ = 10.52, df = 4, *P* < 0.05).

In addition to variation between medical and foundation schools, being UK secondary educated was associated with higher odds of having applied to GP training, while being male, coming from the highest social classes (NS-SEC4/5), being on a graduate entry programme at medical school, or intercalating were associated with lower odds (Table 3).

#### Typologies

The mean predicted probability of having applied solely to GP specialty training for doctors on all entry programmes was 0.26 (Table 2). Irrespective of entry status (SE/GSE/GE), UK secondary school-educated females in social class NS-SEC 1 who had not intercalated at medical school had the highest probability of having applied solely to GP specialty training (SE = 0.35, GSE = 0.40, GE = 0.32) (Additional file 5: Table S5). In contrast, males in social class NS-SEC 5 whose secondary education was outside UK and who had intercalated had the lowest probability (SE = 0.08, GSE = 0.10, GE = 0.08).

**Fig. 5:**
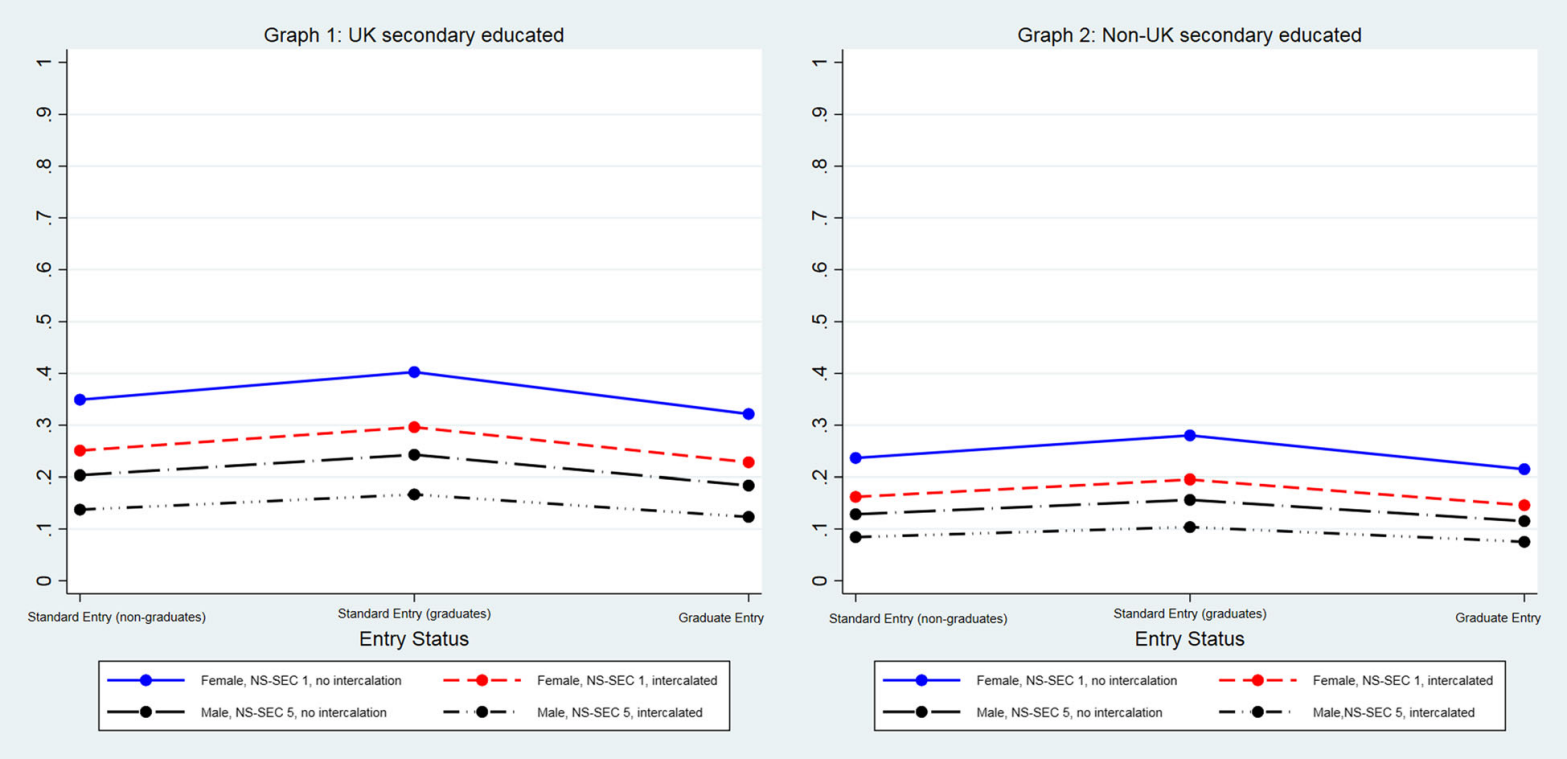
Predicted probability of the outcome ‘applied solely to GP specialty training’ adjusted by entry status, sex, NS-SEC, intercalation and whether a doctor was educated at secondary school level in or out of the UK (Model 3: sample = all entry programmes)

For any given typology, graduate entrants to standard entry programmes had a higher probability of having applied solely to GP specialty training than non-graduate entrants to standard entry programmes and those on graduate entry programmes (Additional file 5: Table S5, Fig. 5 graphs 1 and 2).

### Model 4: Applied solely to GP specialty training (non-graduate entrants to medical school)

Sex, BME, secondary school type, total UKCAT score, intercalation, medical school and foundation school were significantly associated with the likelihood of having applied solely to GP specialty training (Table 2). The interaction of BME and intercalation was significant (χ^2^ = 8.85, df = 1, *P* < 0.01).

In addition to variation between medical and foundation schools, being from a BME background was associated with higher odds of having applied to GP training, while being male, independent school educated, having a high UKCAT score or intercalating during medical school were associated with lower odds (Table 3).

#### Typologies

**Fig. 6:**
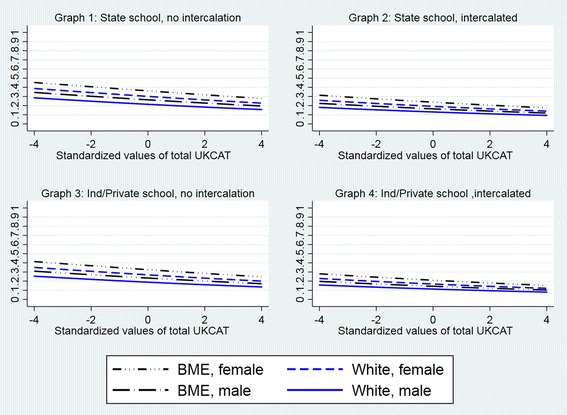
Predicted probability of the outcome ‘applied solely to GP specialty training’ adjusted by sex, BME, type of school attended, intercalation and performance in the UK Clinical Aptitude Test (Model 4: sample = standard entry programmes excluding graduate entrants)

The mean predicted probability of having applied solely to GP specialty training for subjects who had studied medicine as non-graduate entrants was 0.26 (Table 2). For any given typology, based on school type, sex and ethnicity, intercalaters were less likely to have applied solely to GP specialty training than their counterparts who had not intercalated (Additional file 6: Table S6, Fig. 6). Female, BME subjects who had attended a state school but not intercalated had the highest probability (0.36), while male, white, independent school-educated subjects who had intercalated had the lowest probability (0.11) over one standard deviation below the mean.

## Discussion

Our study has identified influential factors that are independently associated with the likelihood of applying to GP specialty for the cohort of junior doctors applying to core training posts in the UK during 2015. Further, it has responded to calls for a clearer insight into the differences between groups, defined by student characteristics in respect of the career choices they make [12]. Overall, 43% of the sample applied to GP specialty training, but only 26% applied as a single specialty application. Significant predictors included individual characteristics (sex, ethnicity, IMD, NS-SEC), educational environment (secondary school type, UK vs. non-UK educated, graduate vs. non-graduate entry, medical school attended, foundation school attended), and measures of prior academic attainment (intercalated degree, EPM, UKCAT score). For all measures of academic attainment, a stronger performance was associated with a decreased likelihood of application to GP training. BME females who had been UK secondary school educated and had not intercalated at medical school were most likely to apply to GP training, whereas white male intercalaters, secondary educated outside the UK were the least likely. Graduate entrants to standard entry programmes were more likely than both non-graduate entrants to standard entry programmes and graduate programme entrants, to apply. Interestingly, age was not a significant independent predictor. There was a major effect due to medical school attended, even after correcting for differences due to sociodemographic and educational factors. When graduate entrants were excluded from the analysis, independent school educated, coming from an area of low deprivation and having a high UKCAT score were additional factors associated with lower odds. Our regression analysis highlighted differences due to foundation school as well as medical school attended, indicating that, even after correcting for medical school attended, the learning environment of deaneries independently influences career decisions. As well as identifying the main effect of individual predictors, our analyses have also defined typologies based on predicted probabilities for specified student characteristics, thereby enabling a much more nuanced insight into which configurations of characteristics influence decisions, for whom and how.

A major strength of our study lies in its utilisation of the UKMED database, which pulls together hitherto disparate datasets held by diverse organisations to create a unified picture of doctors’ pathways from school to specialty training. The database contained data on specialty training applications for a complete cohort of UK graduates, thereby providing an opportunity, previously unattainable in UK medical education research, to simultaneously investigate the possible association of 25 educational and sociodemographic factors with the likelihood of applying to GP training. However, there may have been factors that were not included on the UKMED database, such as marital status, which may have an effect. The novelty of the database also brought some limitations. For example, data was only available for a single cohort/year group of UK medical students and the time window was limited. Given the latter, we analysed first-time applications to specialty training only as the current data does not capture the substantial proportion of doctors who apply to GP training more than 5 years after qualifying [9]. However, such limitations will diminish as the database grows, and our study shows the potential of the UKMED project as an important resource for researchers in this field. Future studies may benefit from the addition of data from more cohorts and greater scope of the included fields. For example, our results show that, even after allowing for demographic factors and measures of educational attainment, the likelihood of applying to GP training varies independently between both medical schools and foundation schools. However, the database currently lacks information that might help explain this variation, such as the extent of clinical exposure to GP experience by medical students or which specialty placements were undertaken during foundation training. However, as previously noted by Davison et al. [9], expanding the coverage of UKMED will take some time and its scope, particularly regarding medical students’ experiences, interests and intentions, will likely remain limited.

Factors associated with specialty choice have previously been classified into five main categories, namely (1) medical school characteristics (e.g. curriculum structure), (2) student characteristics (e.g. age, personality), (3) student values (personal preference), (4) needs to satisfy (expected income, status, work-life balance), and (5) perceptions of specialty characteristics (e.g. extracurricular or curricular experiences) [10]. In the main, extant studies have focussed on categories 3, 4 and 5, providing valuable qualitative insights into the mechanisms and motives that influence specialty choice intentions. A major strength of our study is that it is based not on self-reported intentions but on actual specialty applications of a UK national cohort and thus is not prone to response rate and representativeness bias. Choosing ‘applications to GP training’ as the main outcome variable for our analyses rather than ‘entry into GP training programmes’, meant that our results would not be clouded by the selection-related factors that might prevent applicants from actually entering training. Our study complements existing survey-based evidence revealing a wide disparity in the proportion of graduates from individual medical and foundation schools entering GP training [16, 27]. Our analyses further indicate independent effects due to both medical and foundation school attended that are not solely due to between-school differences in the personal characteristics of their student/trainee cohorts. This finding adds evidence to longitudinal cohort studies assessing ‘attractiveness of GP’ in medical graduates, as reported by Davison et al. [9], who stated that “*career preferences are malleable both during medical school and following graduation*”. However, we were unable to analyse levels of interest in GP amongst study participants, which could explain some of the variation between schools. Our results substantiate the claim, by national reports and student surveys, that differences in career choice are related to variation in the curricula and culture of medical schools [12, 21, 28] and underlines recommendations for further research into those differences [29]. We found a significant effect of ‘intercalation’, which supports findings by Lambert et al. [30] highlighting marked differences between intercalaters (15.3%) and non-intercalaters (25.9%) choosing GP as a career. Querido et al. [10] found that one-third of the studies included in their systematic review of medical student career choices reported sex as a direct determinant. Our study aligns with this general finding of the main effect of sex, but also adds evidence that the influence of sex on career decisions is likely mediated by ethnicity, type of undergraduate degree programme, entry status, aptitude at entry, subsequent academic performance and whether secondary educated in the UK or not.

Identifying factors associated with junior doctors’ decisions to follow particular career pathways is particularly important in light of the growing demand-supply imbalance in the number of GPs available to meet service need in primary care. The fact that females are more likely to apply to GP may mitigate against low numbers of applicants since there are increasing percentages of female graduates [31]. However, numbers of applicants to GP training are not increasing proportionately with feminisation of the workforce since attitudes, values and experience during GP placements are also contributory factors [32]. The widening participation agenda aims to balance the characteristics of the workforce with the patient population it serves, in order to provide the best possible care [33, 34]. Our findings confirm that this agenda should take account of ethnicity, sex, social status and type of secondary school education when developing selection strategies to widen participation in medicine. Using aptitude tests, such as UKCAT, to set thresholds for undergraduate selection may help to open opportunities for under-represented sociodemographic groups [35], but cut scores should not be set so high that candidates selected are more likely to pursue non-GP specialties, since we found an inverse relationship between UKCAT score and likelihood of applying to GP training. Workforce planning strategies should take account of educational factors that affect trainees’ career choices to inform policies for expanding medical student numbers and postgraduate training posts. The UK is increasing medical school numbers by an extra 1500 places in order to improve future service provision [36]; these resources should be targeted at schools that deliver graduates likely to fill significant gaps in the workforce. The Association of American Medical Colleges has responded to projected shortfalls by increasing enrolment of medical students by 30% and expanding graduate medical education programmes [3]. However, our study offers no evidence to support the latter strategy; we found that, among doctors who had entered medical school as graduates, those from graduate entry programmes were no more likely than those from standard programmes to apply for GP training.

Understanding the interactions involved in complex career decision-making processes requires mixed methodological, longitudinal studies of large student cohorts. National datasets such as UKMED provide opportunities to analyse a multitude of factors associated with applications to specific medical careers, successful appointment and subsequent completion of specialty training. We have found significant independent factors associated with applications to GP specialty training related to personal characteristics, educational environment and academic attainment during training. Career choice is a dynamic process; future research should explore how these decisions are affected by individual motives and values, as well as the changing learning environments in which they are made.

## Conclusions

Our study identified factors associated with actual applications to GP training posts in the UK that included ethnicity, sex, UK versus non-UK secondary education, and academic attainment at entry to and across the medical degree programme. Medical school and foundation school attended had a significant effect, even after correcting for educational and sociodemographic factors. Further research is required to understand why these differences exist and to develop strategies to improve recruitment of GP trainees. Our findings will inform policies to widen participation in medicine and improve recruitment of junior doctors to GP training posts, in order to address service need.

## Additional files

Additional file 1: Table S1. Sociodemographic and educational background descriptive statistics of the UKMED sample of doctors who applied for specialty training in 2015. (DOCX 14 kb)

Additional file 2: Table S2. The percentage of doctors who applied to GP specialty training contrasted by medical school and foundation school attended. (DOCX 23 kb)

Additional file 3: Table S3. Typologies derived from Model 1. (DOCX 16 kb)

Additional file 4: Table S4. Typologies derived from Model 2. (DOCX 14 kb)

Additional file 5: Table S5. Typologies derived from Model 3. (DOCX 16 kb)

Additional file 6: Table S6. Typologies derived from Model 4. (DOCX 13 kb)

## Abbreviations

ARCP: Annual Review of Clinical Progression
BME: Black and minority ethnic
EPM: Educational Performance Measure
GE: entrant to graduate entry programme
GP: general practice/practitioner
GSE: graduate entrant to standard entry programme
HESA: Higher Education Statistics Agency
IMD: Index of Multiple Deprivation
NS-SEC: National Statistics Socio-economic Classification
POLAR2: Participation Of Local Areas classification
SE: non-graduate entrant to standard entry programme
UKCAT: UK Clinical Aptitude Test
UKMED: UK Medical Education Database
