# Supplementary material for: Factors associated with junior doctors’ decisions to apply for general practice training programmes in the UK: secondary analysis of data from the UKMED project
Source: BMC Med. 2017 Dec 21;15:220. doi: 10.1186/s12916-017-0982-6 (PMC5738759; doi:10.1186/s12916-017-0982-6)
Supplement: Supplementary file 1 — Sociodemographic and educational background descriptive statistics of the UKMED sample of doctors who applied for specialty training in 2015. (DOCX 14 kb) [file 12916_2017_982_MOESM1_ESM.docx]

Table S1: Socio-demographic and educational background descriptive statistics of the UKMED sample of doctors who applied for specialty training in 2015. Results of bivariate tests of association with the outcome applied to GP specialty training (Fisher’s Exact test, Pearson’s Chi squared test, logistic regression as appropriate) with associated statistics and significance. In compliance with the UK Medical Database Statistical Disclosure Control whole numbers have been rounded to the nearest multiple of five. For a full list of UKMED data types, descriptions and sources please refer to the UKMED Data Dictionary available at <http://www.ukmed.ac.uk/documents/UKMED_data_dictionary.pdf>

| Factor | Category | N doctors | % of sample | % applied GP | | Bivariate association |
| --- | --- | --- | --- | --- | --- | --- |
| Gender | Female | 4435 | 58.08 | 47.54 | | Fisher’s Exact  P<0.001 |
|  | Male | 3200 | 41.92 | | 37.47 |  |
|  |  | 7635 | 100.00 | |  |  |
| Age on entry to medical school | <21 years | 5205 | 68.18 | | 41.02 | X²(2) = 73.52 P<0.001 |
|  | >=21 year & <30 years | 2190 | 28.65 | | 46.18 |  |
|  | >=30 years | 240 | 3.17 | | 66.94 |  |
|  |  | 7635 | 100.00 | |  |  |
| Black and Minority Ethnicity (BME) status | BME | 2250 | 32.07 | | 46.28 | Fisher’s Exact  P<0.001 |
|  | Not BME | 5150 | 67.42 | | 41.89 |  |
|  | Not stated/missing | 40 | 0.51 | | 48.15 |  |
|  |  | 7635 | 100.00 | |  |  |
| Disabled | Yes | 80 | 1.05 | | 36.25 | n/s |
|  | No | 7500 | 98.26 | | 43.42 |  |
|  | Not stated/missing | 55 | 0.69 | | 39.62 |  |
|  |  | 7635 | 100.00 | |  |  |
| Income Support (during school years student lived in household which received income support) | Yes | 885 | 11.62 | | 47.46 | Fisher’s Exact  P<0.05 |
|  | No | 5170 | 67.68 | | 43.45 |  |
|  | Unknown/missing | 1580 | 20.70 | | 40.47 |  |
|  |  | 7635 | 100.00 | |  |  |
| Free School Meals (received free school meals) | Yes | 540 | 7.07 | | 50.19 | Fisher’s Exact  P<0.01 |
|  | No | 5790 | 75.81 | | 43.48 |  |
|  | Unknown/missing | 1310 | 17.12 | | 39.79 |  |
|  |  | 7635 | 100.00 | |  |  |
| Socio-economic class (National Statistics socio-economic five point scale) | Semi-routine and routine occupations | 675 | 8.84 | | 50.96 | X²(4) = 24.60  P<0.001 |
|  | Lower supervisory & technical occupations | 125 | 1.64 | | 41.60 |  |
|  | Small employers & own account work | 345 | 4.52 | | 47.25 |  |
|  | Intermediate occupations | 680 | 8.89 | | 45.21 |  |
|  | Managerial & professional occupations | 5065 | 66.33 | | 41.73 |  |
|  | Unknown/missing | 745 | 9.77 | | 43.97 |  |
|  |  | 7635 | 100.00 | |  |  |
| Index of Multiple Deprivation (IMD) a quintile ranking of IMD zone within country of students’ domicile postcode. | 1 | 2470 | 32.34 | | 41.51 | X²(4) = 32.09  P<0.001 |
|  | 2 | 1710 | 22.36 | | 44.23 |  |
|  | 3 | 1290 | 16.87 | | 45.34 |  |
|  | 4 | 805 | 10.52 | | 47.45 |  |
|  | 5 | 530 | 6.94 | | 54.15 |  |
|  | Unknown/missing | 840 | 10.96 | | 32.86 |  |
|  |  | 7635 | 100.00 | |  |  |
| POLAR2 (quintile classification of areas for young participation rates in higher education based on students’ postcode | Low POLAR2 (quintile 1) | 315 | 4.11 | | 46.13 | X²(2) = 44.27  P<0.001 |
|  | Other POLAR2 (quintiles 2 to 5) | 6535 | 85.62 | | 44.52 |  |
|  | Non-UK | 745 | 9.77 | | 31.90 |  |
|  | Unknown/missing | 40 | 0.5 | | 37.50 |  |
|  |  | 7635 | 100.00 | |  |  |
| UK Country (UK location of students’ IMD zone) | England | 4210 | 55.13 | | 43.24 | n/s |
|  | Scotland | 510 | 6.68 | | 38.82 |  |
|  | Northern Ireland | 320 | 4.15 | | 45.53 |  |
|  | Wales | 195 | 2.54 | | 46.91 |  |
|  | Unknown/missing | 2405 | 31.49 | | 43.84 |  |
|  |  | 7635 | 100.00 | |  |  |
| Secondary school type attended | State funded | 4875 | 63.89 | | 45.68 | X²(2) = 30.87  P<0.001 |
|  | Privately funded | 1625 | 21.3 | | 39.42 |  |
|  | Unknown/missing | 1130 | 14.82 | | 38.73 |  |
|  |  | 7635 | 100.00 | |  |  |
| UK Educated | UK secondary & undergraduate | 6710 | 87.91 | | 44.20 | Fisher’s Exact  P<0.001 |
|  | Neither or either | 810 | 10.61 | | 36.05 |  |
|  | Unknown/missing | 110 | 1.48 | | 43.36 |  |
|  |  | 7635 | 100.00 | |  |  |
| UK Secondary School Educated | Yes | 7070 | 92.59 | | 44.07 | Fisher’s Exact  P<0.001 |
|  | No | 455 | 5.93 | | 31.57 |  |
|  | Unknown/missing | 115 | 1.48 | | 43.36 |  |
|  |  | 7635 | 100.00 | |  |  |
| Parent Degree (parent(s)/guardian completed university degree or equivalent) | Yes | 4135 | 54.13 | | 41.59 | Fisher’s Exact  P<0.001 |
|  | No | 1420 | 18.59 | | 49.20 |  |
|  | Unknown/missing | 2085 | 27.29 | | 38.38 |  |
|  |  | 7635 | 100.00 | |  |  |
| Medical school Entry Status | Non-graduate entrant (standard entry programme | 5540 | 72.57 | | 41.17 | X²(2) = 12.02  P<0.001 |
|  | Graduate entrant to standard entry programme | 950 | 12.41 | | 48.57 |  |
|  | Entrant to graduate entry programme | 1150 | 15.02 | | 49.35 |  |
|  |  | 7635 | 100.00 | |  |  |
| First Medical School | Name (see Table 1S for details) | 7635 | 100.00 | | 43.32 | X²(32) = 164.22  P<0.001 |
| Intercalated | Yes | 880 | 11.54 | | 31.56 | Fisher’s Exact  P<0.001 |
|  | No | 6755 | 88.46 | | 44.85 |  |
|  |  | 7635 | 100.00 | |  |  |
| Foundation Programme Selection Assessment: Educational Performance Measure (EPM) | Deciles 39 to 43 | 3620 | 47.42 | | 40.83 | Fisher’s Exact  P<0.001 |
|  | Deciles 34 to 38 | 3145 | 41.21 | | 45.87 |  |
|  | Unknown/missing | 870 | 11.37 | | 44.47 |  |
|  |  | 7635 | 100.00 | |  |  |
| Foundation School Deanery | Name (see Table 1S for details) | 7570 | 99.16 | | 43.32 | X²(27) = 80.49  P<0.001 |
|  | Unknown/missing | 65 | 0.84 | |  |  |
|  |  | 7635 | 100.00 | |  |  |
| Annual Review of Competence Progression (ARCP) Final Outcome | Satisfactory | 6660 | 87.25 | | 43.84 | Fisher’s Exact  P<0.01 |
|  | All other outcomes* | 910 | 11.91 | | 40.92 |  |
|  | Unknown/missing | 65 | 0.84 | | 23.44 |  |
|  |  | 7635 | 100.00 | |  |  |
| Continuous Variables | |  | Mean (SD)) | |  | Regression |
| Total UCAS tariff for all HESA Tariff included qualifications | | 5395 | 471.02  (112.88) | |  | Β = -0.001  P<0.001 |
| Total score in UK Clinical Aptitude Test  (standardized z-score used in analysis) | | 6490 | 0  (1) | |  | Β = -0.24  P<0.001 |
| Total score in Graduate Medical Schools Admissions Test (GAMSAT) | | 620 | 61.49  (7.11) | |  | n/s |
| Age at entry to medical school | | 7635 | 20.27  (3.64) | |  | Β = -0.24  P<0.001 |
| Foundation Programme Selection Assessment: Situational Judgement Test score | | 3710 | 40.65  (3.26) | |  | n/s |
