# Supplementary material for: Factors associated with junior doctors’ decisions to apply for general practice training programmes in the UK: secondary analysis of data from the UKMED project
Source: BMC Med. 2017 Dec 21;15:220. doi: 10.1186/s12916-017-0982-6 (PMC5738759; doi:10.1186/s12916-017-0982-6)
Supplement: Supplementary file 2 — The percentage of doctors who applied to GP specialty training contrasted by medical school and foundation school attended. (DOCX 23 kb) [file 12916_2017_982_MOESM2_ESM.docx]

*Table S2: The percentage of doctors who applied to GP specialty training contrasted by medical school attended and by Foundation School attended.*

| Medical School | N students | %  Applied | %  Applied  solely | Foundation  Programme  Deanery | N students | %  Applied | %  Applied  solely |
| --- | --- | --- | --- | --- | --- | --- | --- |
| Aberdeen | 205 | 50.73 | 37.54 | Birmingham | 247 | 51.01 | 27.53 |
| Barts | 370 | 54.05 | 32.40 | Black Country/Shropshire | 196 | 46.94 | 27.04 |
| Birmingham | 504 | 44.44 | 25.99 | Coventry/Warwickshire | 147 | 48.30 | 25.17 |
| Bradford | 25 | 60.00 | 52.00 | East Anglian | 328 | 44.79 | 26.99 |
| Brighton | 135 | 44.44 | 25.93 | Hereford/Worcestershire | 87 | 50.57 | 31.03 |
| Bristol | 195 | 37.95 | 23.59 | Leicester/Northamptonshire | 219 | 56.16 | 35.16 |
| Cambridge | 181 | 24.86 | 12.71 | Mersey | 351 | 41.88 | 28.21 |
| Cardiff | 273 | 44.69 | 27.84 | North Central Thames | 257 | 33.85 | 16.73 |
| Dundee | 142 | 39.44 | 21.83 | North East Thames | 290 | 43.79 | 23.79 |
| Edinburgh | 206 | 28.16 | 17.48 | North West Thames | 225 | 32.00 | 16.44 |
| Glasgow | 265 | 43.77 | 27.55 | North Western | 517 | 43.13 | 23.21 |
| Hull York | 162 | 54.94 | 32.10 | North Yorkshire East Coast | 167 | 46.71 | 26.85 |
| Imperial | 221 | 33.03 | 15.38 | Northern | 391 | 39.39 | 21.23 |
| Keele | 182 | 43.41 | 24.18 | Northern Ireland | 295 | 49.66 | 18.03 |
| King’s | 259 | 36.68 | 22.39 | Oxford | 226 | 33.19 | 18.58 |
| Lancaster | 54 | 40.74 | 29.63 | Peninsula | 190 | 43.16 | 31.05 |
| Leeds | 165 | 40.61 | 23.64 | Scotland East Region | 86 | 38.37 | 18.60 |
| Leicester | 297 | 54.88 | 32.32 | Scotland North Region | 146 | 53.42 | 39.73 |
| Liverpool | 365 | 45.75 | 27.67 | Scotland West Region | 375 | 42.40 | 28.53 |
| Manchester | 355 | 42.25 | 23.38 | Severn | 415 | 40.00 | 26.51 |
| Newcastle | 341 | 43.70 | 24.34 | South Thames | 777 | 41.83 | 23.55 |
| Norwich | 211 | 42.65 | 26.07 | South Yorkshire | 168 | 41.67 | 25.60 |
| Nottingham | 456 | 41.67 | 28.73 | Staffordshire | 142 | 51.41 | 30.99 |
| Oxford | 126 | 26.19 | 7.14 | Trent | 394 | 44.16 | 30.20 |
| Peninsula | 199 | 48.24 | 34.67 | Wales | 373 | 47.72 | 29.22 |
| Queen’s | 280 | 49.29 | 17.50 | Wessex | 298 | 39.93 | 27.18 |
| Sheffield | 200 | 45.50 | 29.50 | West Yorkshire | 263 | 46.77 | 30.04 |
| Southampton | 276 | 40.22 | 22.10 | UNKNOWN | 64 | 23.44 | 18.31 |
| St Andrews | 70 | 42.86 | 20.00 | All | 7634 | 43.32 | 25.60 |
| St George’s | 324 | 46.60 | 29.63 |  |  |  |  |
| Swansea | 122 | 51.64 | 31.15 |  |  |  |  |
| UCL | 190 | 27.37 | 15.26 |  |  |  |  |
| Warwick | 278 | 48.20 | 27.07 |  |  |  |  |
| All | 7634 | 43.32 | 25.60 |  |  |  |  |
