# Supplementary material for: Factors associated with junior doctors’ decisions to apply for general practice training programmes in the UK: secondary analysis of data from the UKMED project
Source: BMC Med. 2017 Dec 21;15:220. doi: 10.1186/s12916-017-0982-6 (PMC5738759; doi:10.1186/s12916-017-0982-6)
Supplement: Supplementary file 5 — Typologies derived from Model 3. (DOCX 16 kb) [file 12916_2017_982_MOESM5_ESM.docx]

*Table S5: Typologies derived from model 3, of predicted probability of applied to solely to GP specialty training computed for combinations of values on the predictors Entry Status, NS-SEC, gender, UK Secondary Educated and Intercalation holding all other indicators in the model at their means (n=6177, mean probability =0.25).*

| Entry Status | NS-SEC | Gender | UK Secondary Educated | Intercalated | Predicted Probability | 95% CI | |
| --- | --- | --- | --- | --- | --- | --- | --- |
| Non-graduate entrant to Standard Entry Programme | 5 | Male | Yes | Yes | 0.14 | 0.11 | 0.16 |
|  |  |  |  | No | 0.20 | 0.18 | 0.22 |
|  |  |  | No | Yes | 0.08 | 0.05 | 0.12 |
|  |  |  |  | No | 0.13 | 0.09 | 0.17 |
|  |  | Female | Yes | Yes | 0.20 | 0.16 | 0.23 |
|  |  |  |  | No | 0.28 | 0.26 | 0.30 |
|  |  |  | No | Yes | 0.12 | 0.07 | 0.17 |
|  |  |  |  | No | 0.18 | 0.13 | 0.24 |
|  | 1 | Male | Yes | Yes | 0.18 | 0.14 | 0.22 |
|  |  |  |  | No | 0.26 | 0.22 | 0.30 |
|  |  |  | No | Yes | 0.11 | 0.07 | 0.16 |
|  |  |  |  | No | 0.19 | 0.11 | 0.23 |
|  |  | Female | Yes | Yes | 0.25 | 0.20 | 0.30 |
|  |  |  |  | No | 0.35 | 0.31 | 0.39 |
|  |  |  | No | Yes | 0.16 | 0.10 | 0.23 |
|  |  |  |  | No | 0.24 | 0.16 | 0.31 |
| Graduate entrant to Standard Entry Programme | 5 | Male | Yes | Yes | 0.17 | 0.13 | 0.21 |
|  |  |  |  | No | 0.24 | 0.21 | 0.28 |
|  |  |  | No | Yes | 0.10 | 0.06 | 0.15 |
|  |  |  |  | No | 0.16 | 0.10 | 0.21 |
|  |  | Female | Yes | Yes | 0.23 | 0.18 | 0.29 |
|  |  |  |  | No | 0.33 | 0.29 | 0.37 |
|  |  |  | No | Yes | 0.15 | 0.10 | 0.21 |
|  |  |  |  | No | 0.22 | 0.15 | 0.29 |
|  | 1 | Male | Yes | Yes | 0.22 | 0.16 | 0.28 |
|  |  |  |  | No | 0.31 | 0.25 | 0.36 |
|  |  |  | No | Yes | 0.14 | 0.08 | 0.20 |
|  |  |  |  | No | 0.20 | 0.13 | 0.28 |
|  |  | Female | Yes | Yes | 0.30 | 0.23 | 0.37 |
|  |  |  |  | No | 0.40 | 0.34 | 0.46 |
|  |  |  | No | Yes | 0.20 | 0.12 | 0.28 |
|  |  |  |  | No | 0.28 | 0.19 | 0.37 |
| Graduate Entry Programme | 5 | Male | Yes | Yes | 0.12 | 0.09 | 0.24 |
|  |  |  |  | No | 0.18 | 0.14 | 0.17 |
|  |  |  | No | Yes | 0.08 | 0.04 | 0.11 |
|  |  |  |  | No | 0.12 | 0.07 | 0.16 |
|  |  | Female | Yes | Yes | 0.26 | 0.13 | 0.23 |
|  |  |  |  | No | 0.11 | 0.20 | 0.31 |
|  |  |  | No | Yes | 0.26 | 0.06 | 0.16 |
|  |  |  |  | No | 0.17 | 0.10 | 0.23 |
|  | 1 | Male | Yes | Yes | 0.16 | 0.11 | 0.22 |
|  |  |  |  | No | 0.24 | 0.18 | 0.30 |
|  |  |  | No | Yes | 0.10 | 0.05 | 0.15 |
|  |  |  |  | No | 0.15 | 0.09 | 0.22 |
|  |  | Female | Yes | Yes | 0.23 | 0.16 | 0.30 |
|  |  |  |  | No | 0.32 | 0.25 | 0.39 |
|  |  |  | No | Yes | 0.15 | 0.08 | 0.21 |
|  |  |  |  | No | 0.22 | 0.13 | 0.30 |
