# Supplementary material for: Factors associated with junior doctors’ decisions to apply for general practice training programmes in the UK: secondary analysis of data from the UKMED project
Source: BMC Med. 2017 Dec 21;15:220. doi: 10.1186/s12916-017-0982-6 (PMC5738759; doi:10.1186/s12916-017-0982-6)
Supplement: Supplementary file 6 — Typologies derived from Model 4. (DOCX 13 kb) [file 12916_2017_982_MOESM6_ESM.docx]

*Table S6: Typologies derived from model 4, of predicted probability of applied to solely to GP specialty training computed for combinations of values on the predictors school type, gender, Intercalation and BME holding all other indicators in the model at their means (n=4327, mean probability =0.26).*

| School Type | Gender | Intercalated | BME | Predicted Probability | 95% C.I. | |
| --- | --- | --- | --- | --- | --- | --- |
| Private / Independent | Male | Yes | White | 0.11 | 0.09 | 0.14 |
|  |  |  | BME | 0.15 | 0.11 | 0.18 |
|  |  | No | White | 0.19 | 0.16 | 0.22 |
|  |  |  | BME | 0.24 | 0.20 | 0.27 |
|  | Female | Yes | White | 0.17 | 0.13 | 0.21 |
|  |  |  | BME | 0.21 | 0.17 | 0.26 |
|  |  | No | White | 0.27 | 0.24 | 0.30 |
|  |  |  | BME | 0.33 | 0.28 | 0.37 |
| State | Male | Yes | White | 0.13 | 0.10 | 0.16 |
|  |  |  | BME | 0.17 | 0.13 | 0.20 |
|  |  | No | White | 0.22 | 0.19 | 0.24 |
|  |  |  | BME | 0.27 | 0.23 | 0.30 |
|  | Female | Yes | White | 0.19 | 0.16 | 0.23 |
|  |  |  | BME | 0.24 | 0.19 | 0.29 |
|  |  | No | White | 0.30 | 0.28 | 0.33 |
|  |  |  | BME | 0.36 | 0.32 | 0.40 |
